# Characterization and Evaluation of Ternary Complexes of Ascorbic Acid with γ-Cyclodextrin and Poly(vinyl Alcohol)

**DOI:** 10.3390/ijms21124399

**Published:** 2020-06-20

**Authors:** Phennapha Saokham, Kanokporn Burapapadh, Pitsiree Praphanwittaya, Thorsteinn Loftsson

**Affiliations:** 1 Department of Manufacturing Pharmacy, College of Pharmacy, Rangsit University, Pathum Thani 12000, Thailand; kanokporn.b@rsu.ac.th; 2 Faculty of Pharmaceutical Sciences, University of Iceland, Hofsvallagata 53, IS-107 Reykjavik, Iceland; pip3@hi.is (P.P.); thorstlo@hi.is (T.L.)

**Keywords:** γ-cyclodextrin, poly(vinyl alcohol), ascorbic acid, inclusion complex, antioxidant activity

## Abstract

Ascorbic acid (AA) is a general antioxidant used in aqueous pharmaceutical formulations. However, in aqueous solutions, AA is unstable and easily oxidized when exposed to air, light and/or heat. Cyclodextrins are well known for their ability to form inclusion complexes with various compounds to improve their solubility and stability. Previous studies demonstrate that cyclodextrins preserve the antioxidant capacity of AA but data for γ-cyclodextrin (γCD) have not been reported. Poly(vinyl alcohol) (PVA) is a hydrophilic polymer widely used as a drug matrix in various pharmaceutical fields, but its application for drug stabilization is limited. This study aimed to investigate the protective ability of γCD on AA through the formation of ternary complexes with PVA. Binary (i.e., AA/γCD, AA/PVA and γCD/PVA) and ternary (i.e., AA/γCD/PVA) complexes were first confirmed. It was reported that those complexes were formed through interactions between the heterocyclic ring of AA, hydroxyl group of PVA and hydrophobic cavity of γCD. The hydrodynamic diameter of complexes was then studied. It was found that the diameter of γCD/PVA complexes increased with respect to the concentration of γCD. Higher γCD concentrations also resulted in increasing hydrodynamic diameters of the ternary complex. The presence of AA in ternary complexes interfered with the aggregation tendency of γCD/PVA binary complexes. Furthermore, the antioxidant capacity of AA in binary and ternary complexes was investigated. It was found that the presence of γCD preserved the antioxidant activity of AA, whereas PVA showed a contrasting effect. The influence of γCD and PVA concentration on antioxidant capacity was then studied through central composite design (CCD). Even though the concentration of γCD significantly affected the inhibition efficiency of the ternary complex, the insignificant influence of PVA could not be ignored. A promising protective ternary complex should consist of an optimized concentration of PVA and a high concentration of γCD.

## 1. Introduction

Cyclodextrins (CDs) are cyclic oligomers of six, seven, or eight d-glucopyranose units linked by α-glucosidic bonds. Among the members of natural CDs, γCD has the widest cavity (ca. 0.80–0.95 nm) followed by βCD and αCD (ca. 0.62–0.78 and 0.50–0.57 nm, respectively) [1,2]. Due to their torus shape with a hydrophobic cavity, CDs are widely used to enhance solubility, dissolution, and bioavailability in pharmaceutical applications [3,4]. The ability to form an inclusion complex of CD with a hydrophobic molecule can protect degradation and preserve the activities of labile compounds, such as essential oils [5], volatile oils [6] and antioxidants [2,7,8]. From a stability perspective, the use of only drug/CD inclusion complexes may not be sufficient. Because the inclusion complex is a dynamic process, different parts of a drug molecule could be included in the cavity of CDs resulting in varying molecular conformations. Drug molecules associated with CDs are protected, whereas non-inclusion drugs are degraded. The equilibrium of the inclusion complex also depends on various factors such as the type of CD, pH of the medium, and the presence of additives. Thus, the protective capability of CDs is difficult to predict and uncontrollable. To overcome these issues, the formation of ternary complexes with a water-soluble polymer is proposed. Poly(vinyl alcohol) (PVA) is a well-known hydrophilic water-soluble polymer used in drug delivery systems. Several techniques can be applied to form PVA hydrogels in aqueous solutions, such as photo-cross linking [9], freezing-thawing [10], irradiation [11,12], polymer blending [13,14], and chemical cross-linking [15,16]. PVA is a good candidate for polymer blending due to its high polarity, aqueous solubility, and biodegradable properties. A number of studies have reported inclusion complex formations containing PVA and natural CDs in aqueous solutions [10,13,14,17,18,19]. Those studies present the formation of γCD/PVA inclusion complexes through chemical linking [19] and freezing-thawing [10].

Ascorbic acid (AA) is a labile vitamin that has been studied in several studies due to its instability in aqueous solutions. It is also highly sensitive to heat, alkali, oxygen, light and some traces of metal (e.g., copper and iron [20]). Therefore, it is of interest to investigate stabilizers and optimum concentrations required to stabilize the antioxidant activity of AA. Inclusion complexes with CDs are some of the most popular techniques used in this context. Previous works [21,22,23,24,25,26] demonstrate that AA forms a 1:1 inclusion complex with αCD and βCD as well as with their hydroxypropyl derivatives. As yet, no inclusion complex of AA/γCD has been confirmed, but some studies [27,28] report the formation of γCD with ascorbic acid derivatives. Herein, this research was aimed to evaluate the physical properties and protective abilities of γCD/PVA complexes through the formation of ternary complexes. Ascorbic acid was used as a model labile drug. Binary and ternary complexes containing AA, γCD and PVA prepared by a polymer blending method were characterized by the continuous variation method (Job’s plot), infrared spectroscopy (IR) and nuclear magnetic resonance (NMR) spectrometry. Furthermore, the influence of AA on γCD/PVA inclusion complexes was estimated through hydrodynamic diameter measurement. The antioxidant activity of AA in both binary and ternary inclusion complexes was also investigated.

## 2. Results and Discussion

### 2.1. Characterization of Binary and Ternary Complexes

#### 2.1.1. Stoichiometry of Ascorbic Acid Binary Complexes

**Figure 1:**
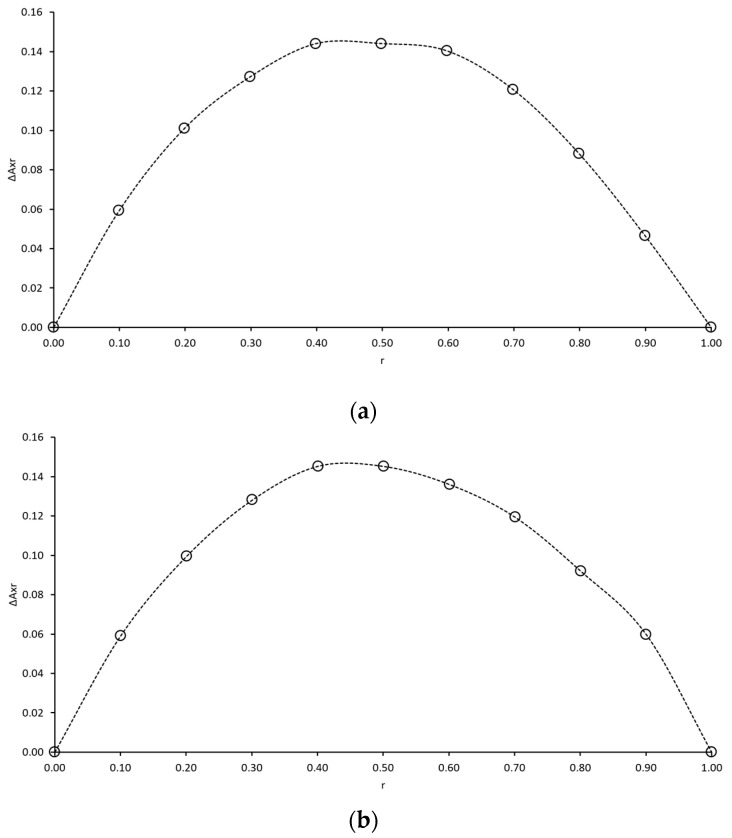
Job’s plots corresponding to the ascorbic acid (AA)/ γ-cyclodextrin (γCD) (**a**) and AA/ Poly(vinyl alcohol) (PVA) (**b**) complexes at λ = 265 nm.

Although various studies confirm that AA forms inclusion complexes with α- and β-cyclodextrin [22,26,29], inclusion complexes between AA and γCD are yet to be verified. The phase solubility technique is well known for drug/CD inclusion complex confirmation. However, in the case of water-soluble drugs such as ascorbic acid, the major disadvantage of this technique is the preparation of saturated AA in γCD solutions. To perform phase solubility studies, saturated AA in various concentrations of γCD should be prepared; hence, a large amount of AA is required. The prepared solutions should also be placed on a horizontal shaker at a constant speed for at least three days to verify that that those solutions are saturated. Because AA is a labile compound that is easily degraded, a preparation of saturated AA in γCD solutions may not be a suitable option. Therefore, the continuous variation technique was proposed. Furthermore, Karim and coworkers [30] demonstrated that PVA can encapsulate AA into its structure so that the formation of AA/PVA complexes could be verified. Considering the maximum of the curve on Job’s plots (Figure 1), the AA/γCD and AA/PVA complexes were confirmed and demonstrated a symmetrical shape with a maximum at r = 0.5 that represented the formation of an equimolar complex (i.e., 1:1, 2:2 or higher n:n, within the investigated concentration range).

#### 2.1.2. Binary and Ternary Complexes in Solid State

Fourier Transform Infrared (FTIR) spectroscopy was used to determine the molecular interaction between two or three molecules, which were binary or ternary complexes, considering the change of intensity and peak in the spectra upon complexation. The low-intensity and very broad band (3000–3600 cm^−1^) with a maximum at 3260.53 cm^−1^ was assigned to the stretching vibration of the O-H groups in the glucose rings of γCD. The C=O and C=C stretching vibration at 1750.89 and 1651.54 cm^−1^, respectively, were assigned to the characteristic bands of the lactone ring of AA [31]. The C-O stretching vibration of PVA was assigned to the moderate-intensity band with a maximum at 1078.72 cm^−1^ [32] (Table 1). The spectra with their characteristic bands are shown in Figures S1–S4 and Table 2, respectively. The similarities between spectra of inclusion complexes and their physical mixtures at a mole ratio of 1:1 and the shifts of the maxima of the characteristic bands were studied.

In the spectrum of the AA/γCD inclusion complex, an increase of intensity and shifting of characteristic bands was observed. The shifts of characteristic groups corresponding to the hydroxyl groups of glucose rings in γCD (45.52 cm^−1^) and lactone ring in AA (10.83 and 37.48 cm^−1^ for carbonyl and alkene groups, respectively) were assumed to be due to the interaction of the lactone ring with hydroxyl groups inside the γCD toroid. The increment of intensity compared to the 1:1 physical mixture indicated that the AA molecule was encapsulated inside the cavity of γCD, resulting in the AA/γCD inclusion complex. In the case of the AA/PVA complex, the disappearance of O-H and C-H alkyl stretching bands (3293.30 and 2914.85 cm^−1^, respectively) shown in Figure S2, along with the shift of C-O stretching vibration band of PVA (7.90 cm^−1^), represented the interaction of the PVA molecule with the carbonyl group in the lactone ring of AA. Furthermore, in the γCD/PVA spectrum, the shift of characteristic bands corresponding to the hydroxyl group of γCD and C-O groups of PVA (60.97 and 1.40 cm^−1^, respectively) and increasing of their intensity indicated the formation of the γCD/PVA inclusion complex. According to the characteristic bands in the spectrum of the AA/γCD/PVA complexes, the dominant shift of the hydroxyl group (46.15 cm^−1^) indicated that the formation of a ternary complex was preferred. In the lactone ring of the AA molecule, the shift of the C=C stretching vibration band was approximately 6.79 times higher than in the binary complexes, whereas the shift of C=O stretching vibration bands was almost identical.

#### 2.1.3. Binary and Ternary Complexes in Aqueous State

**Figure 2:**
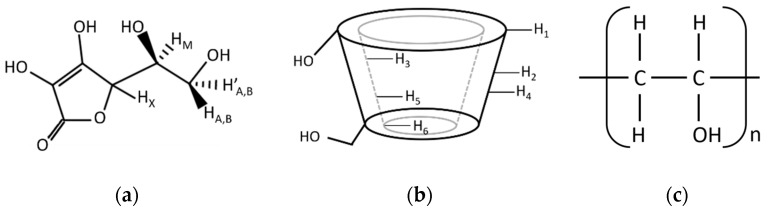
Stereo-chemical configuration of ascorbic acid (**a**), γ-cyclodextrin with internal and external protons (**b**), and poly(vinyl alcohol) (**c**).

The ^1^H NMR spectra of binary and ternary mixtures (Figures S5–S8) were used to support the inclusion phenomena in aqueous solutions. A chemical shift (δ) of protons belonging to AA, γCD, and PVA (Figure 2) was implied in the complexation process. The inclusion complex of γCD (i.e., the host) with AA and PVA (i.e., the guest molecules) was confirmed by the resonance signals of the H_3_ and H_5_ protons located inside the cavity change, which was their chemical shift (Δδ). The magnitude and ratio of ΔδH_3_ and ΔδH_5_ provided information regarding the stability and depth of the inclusion complex. The change of chemical shifts induced on the protons of the AA, γCD, and PVA as a result of the interactions in the binary and ternary complexes are summarized in Table 2. To thoroughly understand those complexes, two-dimensional rotational Overhauser enhancement experiment (ROESY) NMR experiments were performed. The inspection of a ROESY map established a spatial proximity between protons of two and three molecules.

In the AA/γCD spectrum, the interaction between AA and γCD induced upfield shifts of protons located inside of the CD toroid indicated that inclusion complexes of AA/γCD were formed, and this corresponded to the result of the FTIR spectrum. The high value of ΔδH_3_/ΔδH_5_ ratio (1.11) suggested that AA penetrated into the macrocyclic cavity of γCD from the wider rim. The minor upfield of the H_3_ and H_5_ signals showed that weak inclusion complexes were formed, agreeing with the inclusion complexes of AA with αCD and βCD from previous works [22,25]. According to the bidimensional spectrum of the AA/γCD complex (Figure S9), a very weak dipolar correlation supported the formation of a weak complex. In the presence of γCD, the protons of AA, particularly H_x_ upfield shifts, suggested that the lactone ring of the AA molecule was entrapped in the hydrophobic cavity of γCD.

In the ^1^H NMR and 2D ROESY spectra of AA/PVA (Figure S10), the interaction between AA and PVA was confirmed by the absence of H_CH_ and H_OH_ signals and intermolecular cross-peak between H_A,B_ protons of AA and H_CH2_ protons of PVA, respectively. A pronounced upfield shift of H_x_ (−0.0084) indicated that the cyclic lactone structure of the AA molecule, which is rich in π electrons, was associated with the alkyl and hydroxyl groups of PVA. This finding corresponded to the absence of O-H and the notable shift of C-O stretching vibration of PVA in the FTIR spectrum. Furthermore, the inclusion complex of γCD/PVA was observed through the upfield shifts of H_3_ and H_5_ protons and the disappearance of H_CH_ and H_OH_ signals (3.9512 and 3.7716 ppm, respectively) in the ^1^H NMR spectrum. The high value of the ΔδH_3_/ΔδH_5_ ratio (1.21) suggested that PVA penetrated deeply into the toroidal shape of the γCD cavity. The bidimensional spectrum of γCD/PVA (Figure S11) exhibited several intermolecular cross-peaks between protons of γCD (i.e., H_3_ and H_5_) and H_CH2_ protons of PVA demonstrated the inclusion complex of PVA with γCD. Additional dipolar correlations were found between H_CH2_ protons of PVA and other protons in the exterior structure of γCD confirmed that a γCD/PVA non-inclusion complex had formed. Because PVA is a linear chain polymer, it was possible that the long chain of PVA threaded through the γCD hydrophobic cavity at the same time as the polymer swathed around the external surface.

According to the ^1^H NMR spectrum, the formation of AA/γCD/PVA ternary inclusion complexes was confirmed by the deviation of chemical shifts assigned to AA and γCD molecules. A pronounce upfield shift of H_X_ compared to H_M_ and H_A,B_ indicated that the cyclic lactone part of AA preferably interacted with the other two molecules. The absence of H_CH_ and H_OH_ signals also confirmed that the PVA molecule associated with AA and γCD. The high ratio of ΔδH_3_/ΔδH_5_ (1.22) suggested that AA and/or PVA penetrated through the γCD cavity. Several intermolecular cross-peaks, shown in Figure S12, between H_CH2_ protons of PVA and assigned protons in AA and γCD (i.e., H_A,B_, H_M_, H_3_, H_5_, H_2_, and H_4_), exhibited the formation of AA/γCD/PVA ternary complexes.

The results from ^1^H NMR and 2D ROESY spectra agreed with the FTIR spectra and confirmed the formation of binary and ternary inclusion complexes. It is worth noting that the conformation of the ternary complex might be different from those of binary complexes. The conformation of AA/γCD/PVA ternary complexes should be verified by molecular modeling stimulation in future research. Moreover, even though the chemical shifts from proton NMR can be used to confirm complexes, in this case, the carbon-13 NMR spectroscopy can be performed for more information on the intermolecular interaction.

### 2.2. Hydrodynamic Diameter of Binary and Ternary Complexes

**Figure 3:**
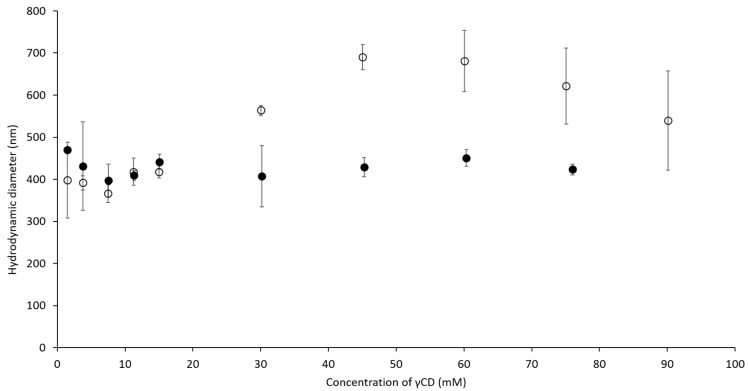
Hydrodynamic diameters of self-assembled γCD molecule (○) and AA/γCD inclusion complex in aqueous solutions (●).

**Figure 4:**
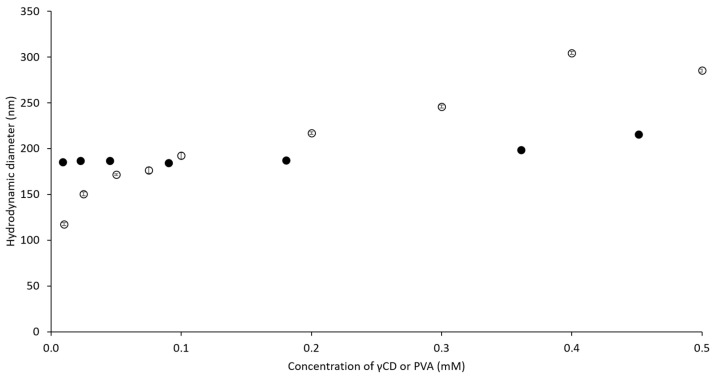
Hydrodynamic diameters of γCD/PVA inclusion complexes at various concentrations of γCD (○) and PVA (●).

To understand the influence of ascorbic acid (AA), γCD, and PVA on the hydrodynamic diameter of complexes, a dynamic light scattering (DLS) technique was used. In Figure 3, the hydrodynamic diameter of self-assembled γCD molecules increased (from 400 to 700 nm) with higher γCD concentrations. When the concentration of γCD was more than 45 mM, haze precipitation was observed at the bottom of the measured cuvette, resulting in a decrease of the obtained diameter. This observation indicated that the self-assembled γCD formed aggregates that then precipitated to the bottom of the cuvette. Compared to the self-assembled γCD molecules, AA/γCD inclusion complexes were recognized when the concentration of γCD was higher than 15 mM. When the concentration of γCD increased further, the diameters of the AA/γCD inclusion complexes were approximately 400–500 nm, and it was assumed that the concentration of γCD did not influence the hydrodynamic diameters of AA/γCD complexes when the concentration of AA was fixed. Furthermore, the influence of γCD and PVA on the hydrodynamic diameter of γCD/PVA inclusion complexes was studied. The diameters of the γCD/PVA inclusion complex increased from 100 to 300 nm (Figure 4, open circle) when the concentration of γCD increased, and PVA was fixed at 0.1 mM. The presence of PVA slightly affected on the hydrodynamic diameter when the concentration of γCD was fixed at 15 mM (Figure 4, closed circle), resulting in an approximate diameter of γCD/PVA inclusion complexes of approximately 180 nm. According to the influence on AA/γCD and γCD/PVA inclusion complexes, it could be assumed that the variation of γCD concentration affected on the hydrodynamic diameter of those two complexes. Thus, the concentrations of AA and PVA were fixed at 0.5 and 344 mM, respectively, for further study on ternary complexes. Furthermore, due to the significant decreasing of hydrodynamic diameters, it could be implied that the formation of AA/γCD and γCD/PVA inclusion complexes interfered with the aggregation tendency of self-assembled γCD in aqueous solutions in accordance with findings for inclusion complexes of γCD with poorly water-soluble drugs [33,34].

**Figure 5:**
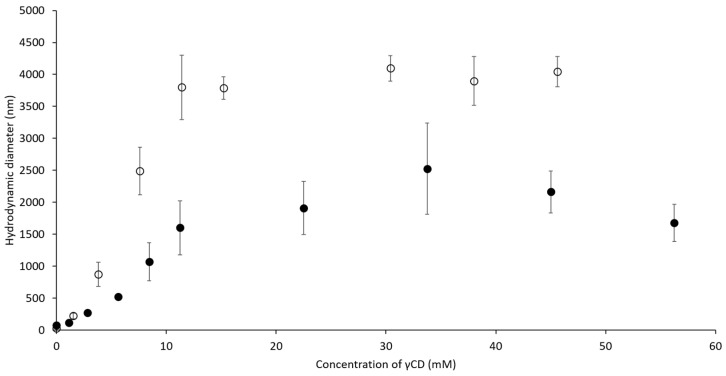
Hydrodynamic diameters of γCD/PVA binary complex (●) and AA/γCD/PVA ternary complex (○) at various concentrations of γCD.

Figure 5 shows that the hydrodynamic diameters of γCD/PVA inclusion complexes were notably smaller than that of the ternary complex. The diameter of the binary complex increased up to 2500 nm upon the increase of the γCD concentration. As in the case of the AA/γCD inclusion complex, a precipitation of aggregates was observed when the concentration of γCD was higher than 34 mM. The increased hydrodynamic diameters as the concentration of γCD also increased were recognized for the AA/γCD/PVA ternary complexes. Their diameters increased up to 4000 nm and then remained stable when the concentration of γCD was higher than 15 mM. This observation indicated that the presence of AA affected the hydrodynamic diameter and aggregation tendency of the γCD/PVA inclusion complex. The hydrodynamic diameter of the AA/γCD/PVA ternary complex remained unchanged throughout storage.

### 2.3. Inhibition Efficiency of Ascorbic Acid in Ternary Complex

**Figure 6:**
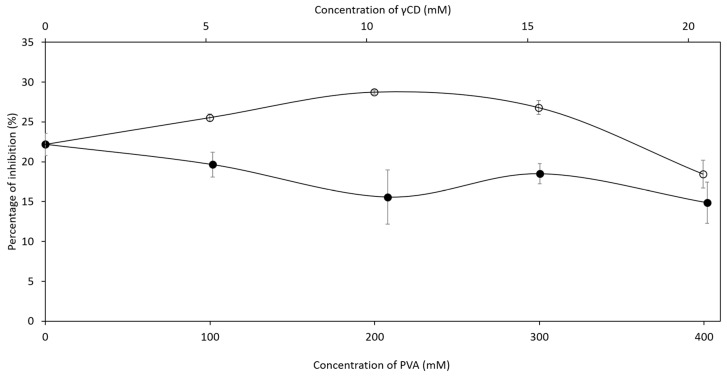
Percentage of inhibition of ascorbic acid in AA/γCD (○) and AA/PVA (●) complexes.

**Figure 7:**
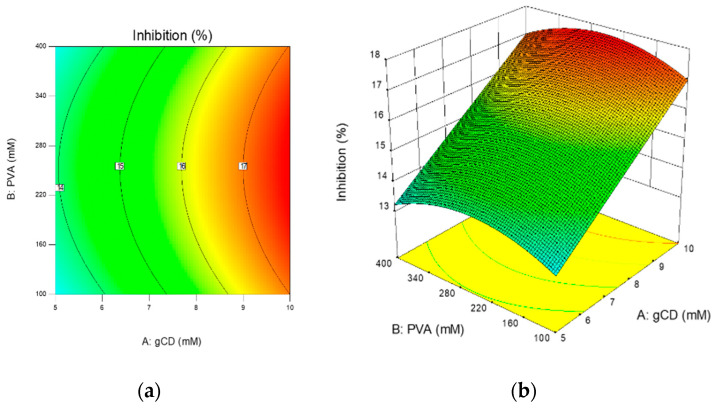
Contour (**a**) and surface (**b**) plots demonstrating relationship between γCD and PVA concentrations on percentage of inhibition.

In pharmaceutical applications, ascorbic acid (AA) is known as an antioxidant that reduces reactive oxygen species, one of the reactive impurities of pharmaceutical ingredients. Because AA was easily oxidized at the hydroxyl groups connected with the C=C of the lactone ring to form dehydroascorbic acid (DHA) [35], the antioxidant activity of those ternary complexes should be investigated. The individual effects of γCD and PVA on inhibition efficiency of AA, representing the active concentration of AA, were investigated prior to experimental design performance to determine the concentration range of the design space. As shown in Figure 6, the presence of γCD in aqueous AA solutions significantly increased the percentage of inhibition, and it was assumed that γCD was able to protect the degradation of AA in aqueous solutions via inclusion complex formation. In contrast to γCD, the inhibition ability of AA decreased with increasing PVA concentration, indicating that the AA/PVA complex was unable to diminish the oxidation reaction of AA in aqueous solutions. For further understanding, the concentrations of γCD and PVA (5–10 and 100–400 mM, respectively) were selected as factors in central composite design (CCD). Thirteen ternary complexes were prepared by designed concentrations of γCD and PVA, as shown in Table 3. The percentage of inhibition was chosen as a response. Analysis of variance (ANOVA) analysis of the quadratic model (R^2^ = 0.8124) in Table 4 indicated that the concentration of γCD significantly affected the inhibition efficiency of AA in the ternary complex. The increase of γCD concentration in the ternary complex resulted in a higher inhibition activity; therefore, the stability of AA in aqueous solutions increased. Even though the concentration of PVA affected to a lesser degree the inhibition efficiency (*p*-value < 0.10), the presence of PVA could not be neglected. According to contour and surface plots (Figure 7), the concentration of PVA should be optimized to maximize the protection ability of the γCD/PVA complex. Many previous studies have demonstrated that βCD can form transient inclusion complexes with a molecule of ascorbic acid to delay the degradation process from oxidation.

## 3. Materials and Methods

### 3.1. Characterization of Binary and Ternary Complexes

#### 3.1.1. Stoichiometry Determination

The stoichiometry of ascorbic acid complexes (such as AA/γCD and AA/PVA binary complexes) was obtained by using the continuous variation technique (i.e., Job’s method, based on the induced change of spectrophotometric absorbance (A)). Aqueous solutions containing 70 mM of AA and γCD were prepared. After they had completely dissolved, those solutions were filtered through a 0.45 µm membrane (VertiPure^®^ nylon syringe filter, Ligand Scientific, Bangkok, Thailand). To determine the stoichiometry of the AA/γCD binary complex, two solutions—aqueous AA and γCD solutions—were mixed in a manner such that the total concentration remained constant, and the molar fraction (r) of AA varied in the range of 0 to 1. The absorbance of AA in mixture solutions was then measured at 265 nm, which was the maximum absorbance of the scanning spectrum from 200 to 400 nm (Evolution™201 UV-Visible spectrophotometer, ThermoFisher Scientific, MA, USA). The deviation of absorbance (ΔA) in the presence of γCD was plotted against the molar fraction. The value of the molar fraction presenting the maximum deviation provided the stoichiometry of the inclusion complex. In the case of 1:1, 2:2, and higher n:n complexes, the maximum deviation is 0.5, whereas the maximum reached 0.37 for the 1:2 complex [36]. For AA/PVA binary complexes, 70 mM of AA and PVA aqueous solutions were used to prepare tested solutions.

#### 3.1.2. Fourier Transform Infrared (FTIR) Spectroscopy

The binary and ternary complexes were prepared at a 1:1 molar ratio (or 1:1:1, in the case of ternary complexes). Ninety mM of AA, γCD and PVA solutions were prepared separately in distilled water and filtered through a 0.45 µm nylon membrane before use. The same volume of two or three solutions was mixed in amber vials closed with a slotted rubber stopper. Complex solutions were then kept in the freezer (Scancool Snowbird Ultra-Freezer, Labogene, Denmark) at −75 °C for 12 h before lyophilization (Alpha 2-4 LCSplus, Christ, Germany) for at least 48 h. The solid complexes were stored in a desiccator until further use. Moreover, the physical mixture of complexes was prepared by mixing an equimolar mass of free compounds (e.g., AA and γCD for AA/γCD binary complex) in a porcelain mortar. Those free compounds were individually passed through an 80 mesh-sieve prior to weighing. The infrared spectra of free compounds (AA, γCD and PVA), physical mixtures, binary and ternary complexes were carried out using a Nicolet 6700 FTIR with an Attenuated Total Reflectance (ATR) attachment (Thermo Scientific, USA). The IR beam was employed from a diamond crystal technique. Each spectrum was measured in the range of 650–4000 cm^−1^ by using OMNIC FT-IR Software, version 9.2.86, Thermo Electron Corporation, USA. The observed FTIR peaks were assigned based on the structure of free compounds (Figure 1). The peaks of complexes were compared with those of physical mixtures. Intramolecular interactions were then exhibited as the deviation of wavenumber (Δcm^−1^). The results represented the formation of binary and ternary complexes in solid form.

#### 3.1.3. Proton Nuclear Magnetic Resonance (^1^H-NMR) Analysis

To investigate complex formation in the liquid state, the ^1^H-NMR spectra were recorded at 500 MHz (AVANCE NEO, Bruker, Switzerland). Approximately 10 mg of free compounds and solid complexes from FTIR studies were dissolved in 0.7 mL of deuterium. The resonance at 7.4 ppm was used as reference to report the chemical shift (δ) values. The changes in chemical shift (Δδ) were calculated according to Equation (1)

(1) $$\Delta \delta ={\;\delta }_{\mathrm{complex}}-{\;\delta }_{\mathrm{free}}$$

Furthermore, the intermolecular interactions were determined by using a rotational Overhauser enhancement experiment (ROESY). The 2D ROESY spectra were collected with a mixing time of 150 ms. The spectrum was acquired with 32 scans. The chemical shift values and intermolecular cross-peaks were investigated using TopSpin™ version 4.0.3 (Bruker, Billerica, MA, USA).

### 3.2. Dynamic Light Scattering (DLS) Analysis

The hydrodynamic diameters of free compounds and complexes in aqueous solution were determined by a particle analyzer (NanoPlus-3, Micromeritics Inc., USA). Stock solutions of free compounds (100 mM) were individually prepared and filtered through a 0.45 µm nylon membrane prior to mixing. First, the effect on binary complexes was investigated. To study the effect of γCD, the hydrodynamic diameters of aqueous γCD solutions, ranging from 1.5 to 90 mM, were measured and compared to solutions of γCD (0–76 mM) that were mixed with 0.52 mM of AA (i.e., AA/γCD complex). In the case of the γCD/PVA complex, the influence of γCD and PVA concentration was determined. The γCD/PVA complexes were prepared in a manner such that the concentrations of γCD were varied, whereas the PVA concentration was constant, and vice versa. Finally, to investigate the hydrodynamic diameters of AA/γCD/PVA ternary complexes, the concentrations of γCD were varied up to 46 mM, and the concentrations of AA and PVA were fixed at 0.52 and 344 mM corresponding to 0.01 and 1.50% *w*/*v*, respectively. Each sample was prepared independently, in triplicate, in a 4-mL polystyrene cuvette (Bioscan, USA) with a silicone cap (Thomas Scientific, USA). The average and standard deviations for the obtained hydrodynamic diameters were then calculated from sextuplicate measurements.

### 3.3. Antioxidant Capacity Studies

DPPH radical-scavenging activities were carried out to investigate the antioxidant capacity of AA in AA/γCD, AA/PVA, and AA/γCD/PVA complexes. An 80 µM methanolic DPPH solution was used as a stock solution. Solutions containing binary complexes consisting of 0.02 mM of AA, 5–20 mM of γCD, and 100–400 mM of PVA were prepared. The DPPH radical was then mixed with free AA or complexes at a volume ratio of 1:2. The reaction of each sample was performed in triplicate in polystyrene cuvettes covered with silicone caps and store in a Styrofoam box at room temperature for 30 min. The decrease in DPPH radical was monitored by measuring the absorbance at a wavelength of 517 nm in a UV-Vis spectrophotometer. The percentage of inhibition (%Inhibition) was calculated by using following equation:

(2) $$\%\mathrm{Inhibition}=\frac{\left(A_{0}-A\right)}{A_{0}}\times 100$$

where $A_{0}$ is the absorbance of the positive control, and A is the absorbance of the sample.

The %Inhibition trend of binary complexes with respect to the concentration of γCD and PVA was investigated, and the concentration ranges for the experimental design were assigned.

### 3.4. Experimental Design

Central composite design (CCD) was chosen to determine the effect of γCD and PVA on the antioxidant capacity of AA/γCD/PVA ternary complexes. Based on the results from antioxidant activity, the concentrations of γCD and PVA were selected as factors, and %Inhibition was the response of the design. To maintain rotability, the α was equal to 1.414. Finally, ternary complexes consisting of assigned concentrations of γCD and PVA were prepared. The concentration of AA for all designed ternary complexes was fixed at 0.02 mM. The percentage of inhibition was then determined (in triplicate for each designed ternary complex) as described in antioxidant capacity studies. The influence of γCD and PVA concentrations on the antioxidant capacity of AA in ternary complexes was determined using a fitted model.

### 3.5. Statistical Analysis

All hydrodynamic diameter and %Inhibition data are presented as average and standard deviation (SD). According to the experimental design, CCD was constructed using statistical software (Design-Expert^®^, version 10.0.5.0, Stat-Ease, Inc., Minneapolis, MN, USA). The quadratic model was firstly fitted and reduced to a two-factor interaction and linear models corresponding to the significant *p*-value (< 0.05) and insignificant Lack-of-fit (*p*-value > 0.05) from analysis of variance (ANOVA). The significant model was accepted when the *p*-value of the model was less than 0.05, with a high goodness of fit (R^2^), and no correlation in the residual plots and the residuals were normally distributed.

## 4. Conclusions

Binary and ternary complexes of ascorbic acid, γCD, and PVA in both solid and liquid states were confirmed in this study via the continuous variation method and spectroscopy (FT-IR and NMR) techniques. The hydrodynamic diameters of binary complexes were approximately 180–2500 and 400–700 nm for γCD/PVA and AA/γCD, respectively. There was an increase of diameter associated with the higher concentrations of γCD incorporated in those complexes. Aggregates and precipitated clusters were observed at high γCD concentrations. Even though the γCD concentration also affected the hydrodynamic diameters of the AA/γCD/PVA ternary complex, those ternary complexes showed less aggregation than those of binary complexes. The presence of AA in γCD/PVA inclusion complexes affected the aggregation tendency of such inclusion complexes. The hydrodynamic diameter of γCD/PVA complexes was stable due to the presence of AA. The protective ability of the γCD/PVA binary complex was then studied via the inhibition efficiency of AA. Increasing the γCD concentration in the ternary complex resulted in a higher inhibition ability of AA. It could be assumed that the γCD/PVA complex was able to protect AA from the oxidation reaction in aqueous solutions when the optimum concentrations of both γCD and PVA were used. In conclusion, this research verifies that PVA molecules can interfere with the precipitation of nano-sized ascorbic acid/γCD inclusion complexes through the formation of ternary inclusion complexes. Even the hydrodynamic diameter of the ternary complexes was larger than the nano-size particles of ascorbic acid/γCD inclusion complexes. Those ternary complexes also showed a protective ability by maintaining the antioxidative activity of ascorbic acid. This finding might lead to a promising application for labile-active pharmaceutical ingredients through ternary complex formation.

## Figures and Tables

**Table 1:** Band assignments of ascorbic acid (AA), γCD, and PVA in binary and ternary complexes.

Assignment	AA	γCD	PVA	Δcm^−1^			
				AA/γCD	AA/PVA	γCD/PVA	AA/γCD/PVA
O-H stretching	-	3260.53	-	45.52	-	60.97	46.15
C=O stretching	1750.80	-	-	10.83	2.69	-	7.76
C=C stretching	1651.54	-	-	37.48	0.63	-	37.46
C-O stretching	-	-	1078.72	-	7.90	1.40	0.77

**Table 2:** Chemical shifts of ascorbic acid (AA), γCD, and PVA in binary and ternary complexes.

Assignment	δ (ppm)	Δδ (ppm)			
		AA/γCD	AA/PVA	γCD/PVA	AA/γCD/PVA
H_X_	4.8658	−0.0073	−0.0084	-	−0.0144
H_M_	3.9977	−0.0027	−0.0013	-	−0.0028
H_A,B_	3.6667	−0.0018	−0.0005	-	−0.0013
H_3_	3.8598	−0.0030	-	−0.0023	−0.0022
H_5_	3.7970	−0.0027	-	−0.0019	−0.0018
H_CH2_	1.6291	-	0.0003	−0.0007	−0.0001

**Table 3:** Central composite design for AA/γCD/PVA ternary complexes when concentration of AA was fixed at 0.02 mM.

Experimental No.	Concentration of γCD (mM)	Concentration of PVA (mM)
1	5	400
2	5	100
3	7.5	50
4	3	250
5	12	250
6	7.5	250
7	7.5	450
8	10	100
9	7.5	250
10	7.5	250
11	7.5	250
12	10	400
13	7.5	250

**Table 4:** Analysis of variance (ANOVA) of quadratic, two-factor, and linear models for the percentage of inhibition.

	Quadratic Model (R^2^ = 0.8124)		Two-Factor Model (R^2^ = 0.6685)		Linear Model (R^2^ = 0.6507)	
	Coefficient	*p*-Value	Coefficient	*p*-Value	Coefficient	*p*-Value
Model	-	0.0175 *	-	0.0153 *	-	0.0052 *
γCD	1.94	0.0015 *	1.89	0.0023 *	1.89	0.0015 *
PVA	0.0277	0.9465	0.0167	0.9723	0.0069	0.9882
γCD*PVA	−0.4462	0.4440	−0.4482	0.5047	-	-
(γCD)^2^	−0.5900	0.1744	-	-	-	-
(PVA)^2^	−0.8360	0.0914 **	-	-	-	-
Lack of Fit	-	0.4871	-	0.3241	-	0.3660

* *p*-value < 0.05 indicated model or terms were significant at 95% confidential interval. ** *p*-value < 0.10 indicated model or terms were significant at 90% confidential interval.

## Supplementary Materials

The following are available online at https://www.mdpi.com/1422-0067/21/12/4399/s1, Figure S1: ATR-FTIR spectra of (a) ascorbic acid, (b) γCD, (c) physical mixture of ascorbic acid and γCD and (d) ascorbic acid/γCD complexes, Figure S2: ATR-FTIR spectra of (a) ascorbic acid, (b) PVA, (c) physical mixture of ascorbic acid and PVA and (d) ascorbic acid/PVA complexes, Figure S3: ATR-FTIR spectra of (a) γCD, (b) PVA, (c) physical mixture of γCD and PVA and (d) γCD/PVA complexes, Figure S4: ATR-FTIR spectra of (a) ascorbic acid, (b) γCD, (c) PVA, (d) physical mixture of ascorbic acid, γCD and PVA and (e) ascorbic acid/γCD/PVA complexes, Figure S5: ^1^H NMR spectra of (a) ascorbic acid, (b) γCD and (c) ascorbic acid/γCD inclusion complexes in D_2_O, Figure S6: ^1^H NMR spectra of (a) ascorbic acid, (b) PVA and (c) ascorbic acid/PVA inclusion complexes in D_2_O, Figure S7: ^1^H NMR spectra of (a) γCD, (b) PVA and (c) γCD/PVA inclusion complexes in D_2_O, Figure S8: ^1^H NMR spectra of (a) ascorbic acid, (b) γCD (c) PVA and (d) ascorbic acid/γCD/PVA complexes in D_2_O, Figure S9: Rotating overhauser effect spectroscopy (ROESY) pattern of ascorbic acid/γCD complexes, Figure S10: Rotating overhauser effect spectroscopy (ROESY) pattern of ascorbic acid/PVA complexes, Figure S11: Rotating overhauser effect spectroscopy (ROESY) pattern of γCD/PVA complexes, Figure S12: Rotating overhauser effect spectroscopy (ROESY) pattern of ascorbic acid/γCD/PVA ternary complexes.

## Abbreviations

AA	Ascorbic acid
ANOVA	Analysis of variance
CCD	Central composite design
FT-IR	Fourier-transform infrared spectroscopy
γCD	γ-cyclodextrin
NMR	Nuclear magnetic resonance
PVA	Poly(vinyl alcohol)
SD	Standard deviation
