# Supplementary material for: Characterization and Evaluation of Ternary Complexes of Ascorbic Acid with γ-Cyclodextrin and Poly(vinyl Alcohol)
Source: Int J Mol Sci. 2020 Jun 20;21(12):4399. doi: 10.3390/ijms21124399 (PMC7352738; doi:10.3390/ijms21124399)
Supplement: Supplementary file 1 [file ijms-21-04399-s001.pdf]

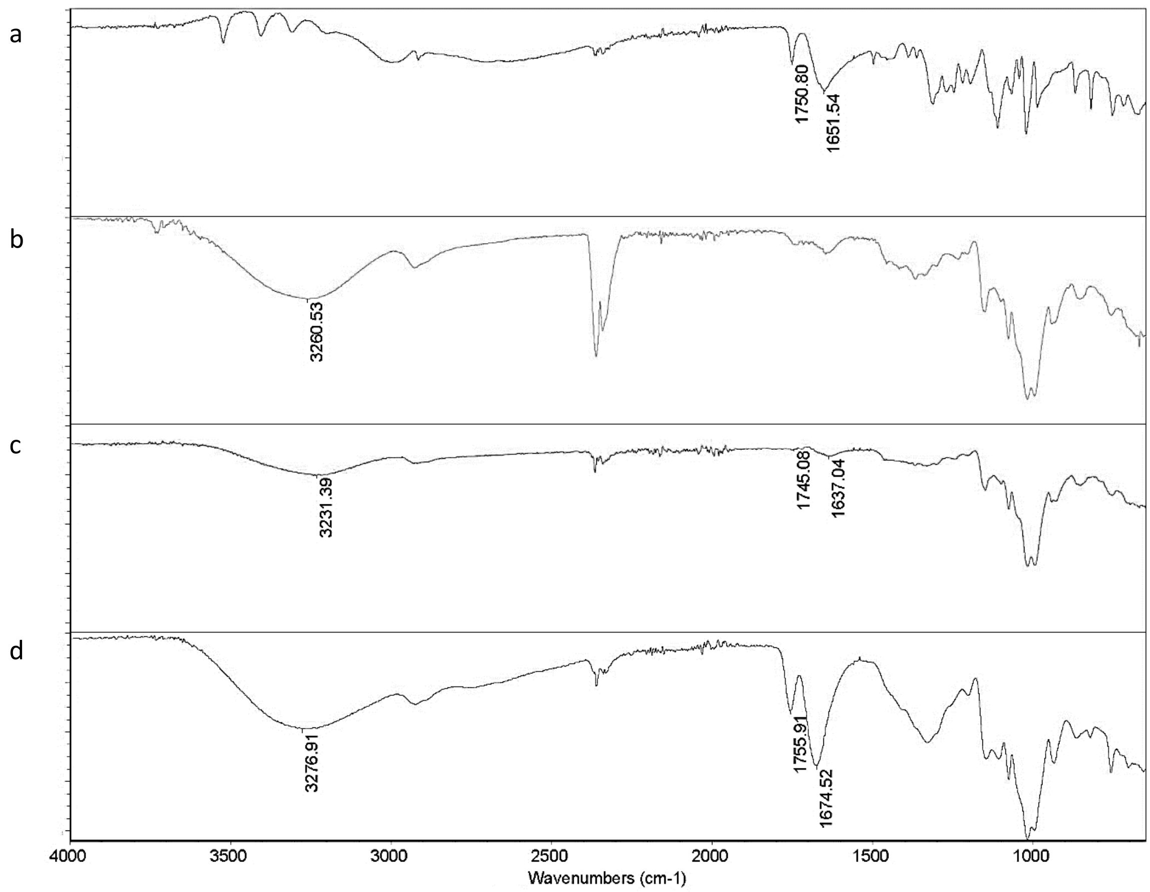

Figure S1 ATR-FTIR spectra of (a) ascorbic acid, (b) γCD, (c) physical mixture of ascorbic acid and γCD and (d) ascorbic acid/γCD complexes.

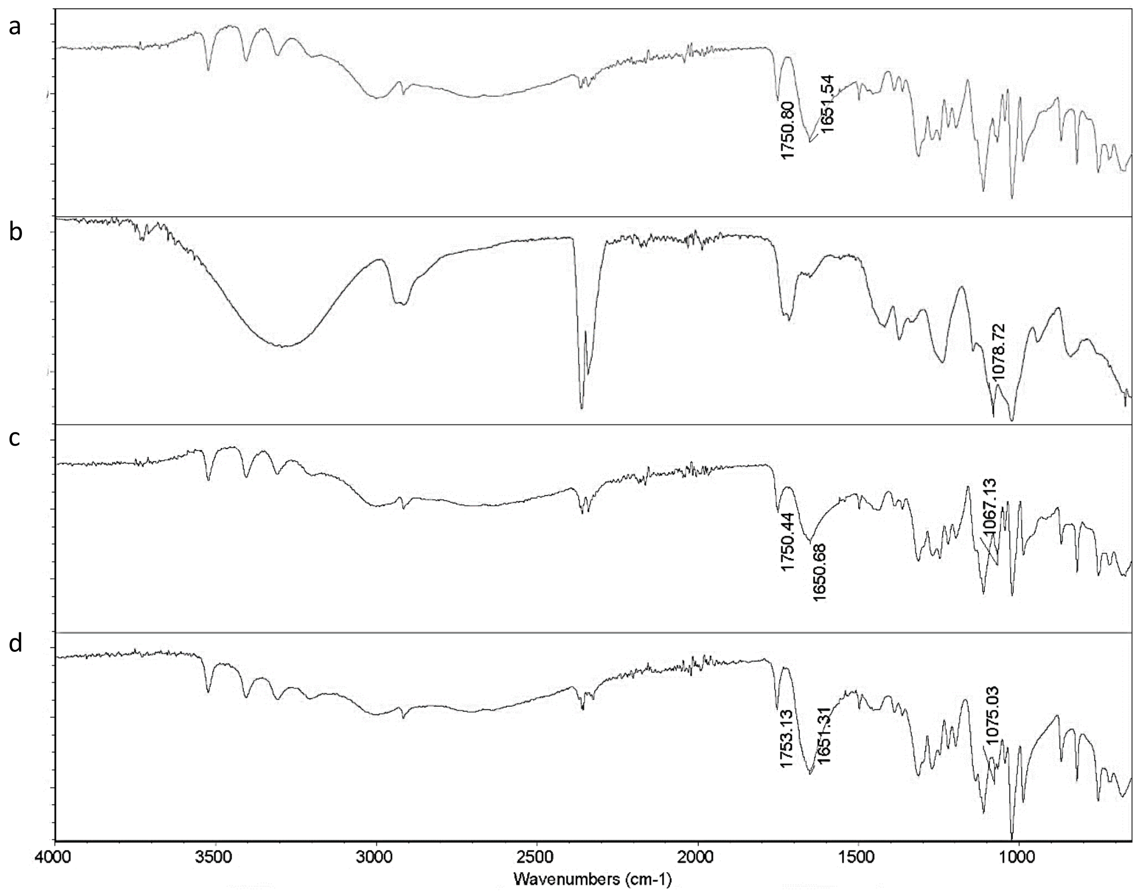

Figure S2 ATR-FTIR spectra of (a) ascorbic acid, (b) PVA, (c) physical mixture of ascorbic acid and PVA and (d) ascorbic acid/PVA complexes.

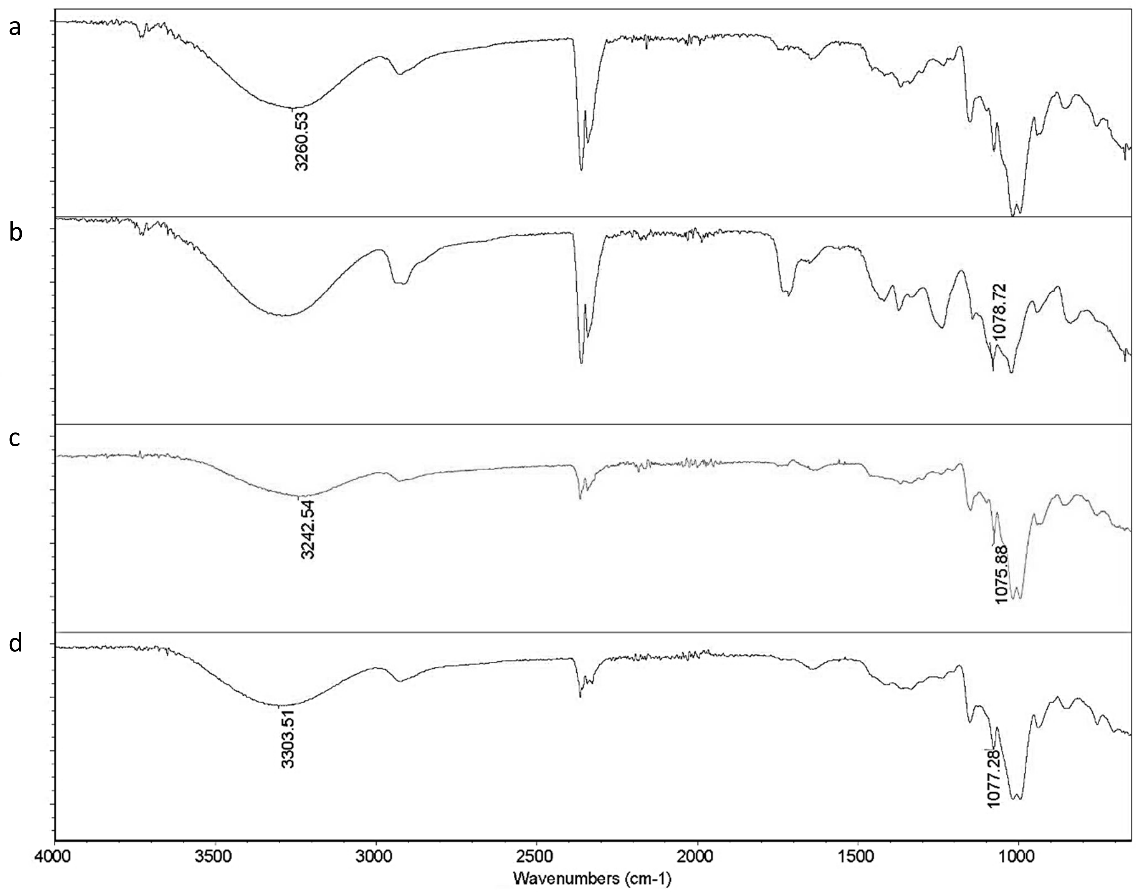

Figure S3 ATR-FTIR spectra of (a) γCD, (b) PVA, (c) physical mixture of γCD and PVA and (d) γCD/PVA complexes.

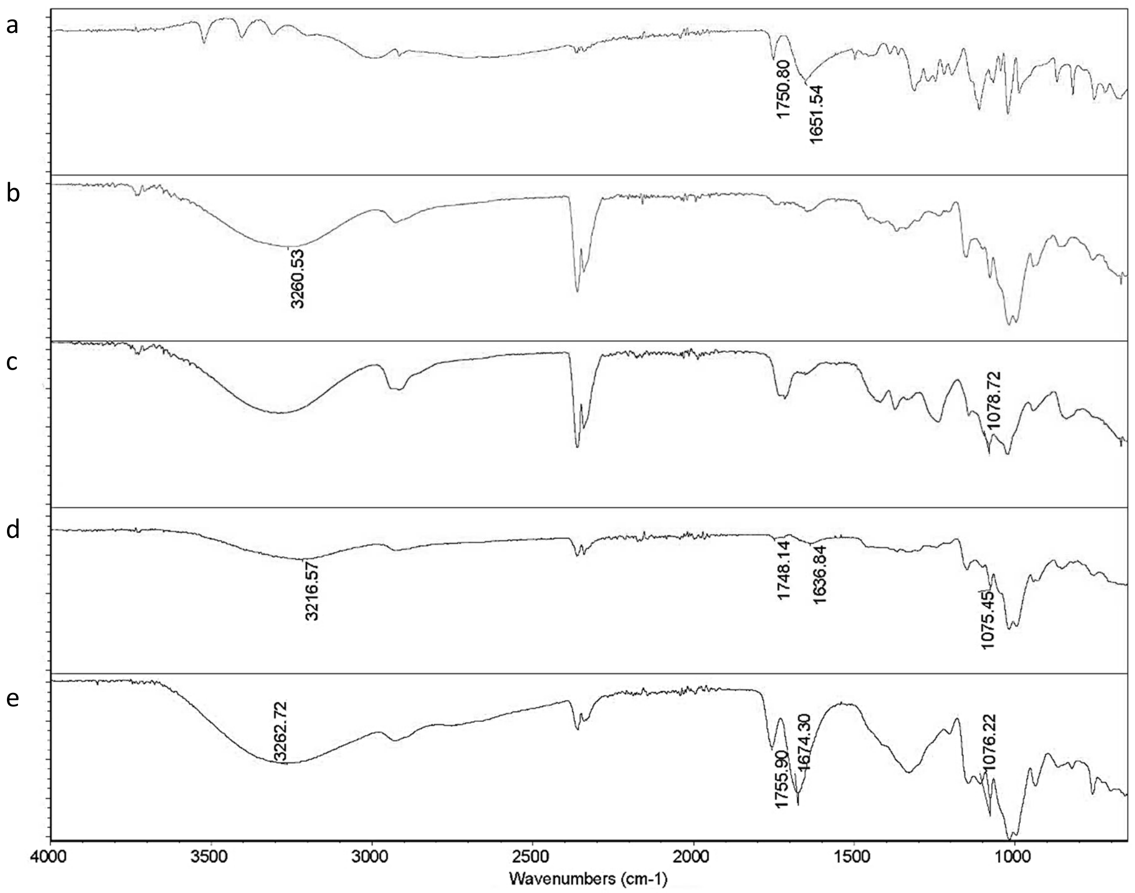

Figure S4 ATR-FTIR spectra of (a) ascorbic acid, (b) γCD, (c) PVA, (d) physical mixture of ascorbic acid, γCD and PVA and (e) ascorbic acid/γCD/PVA complexes.

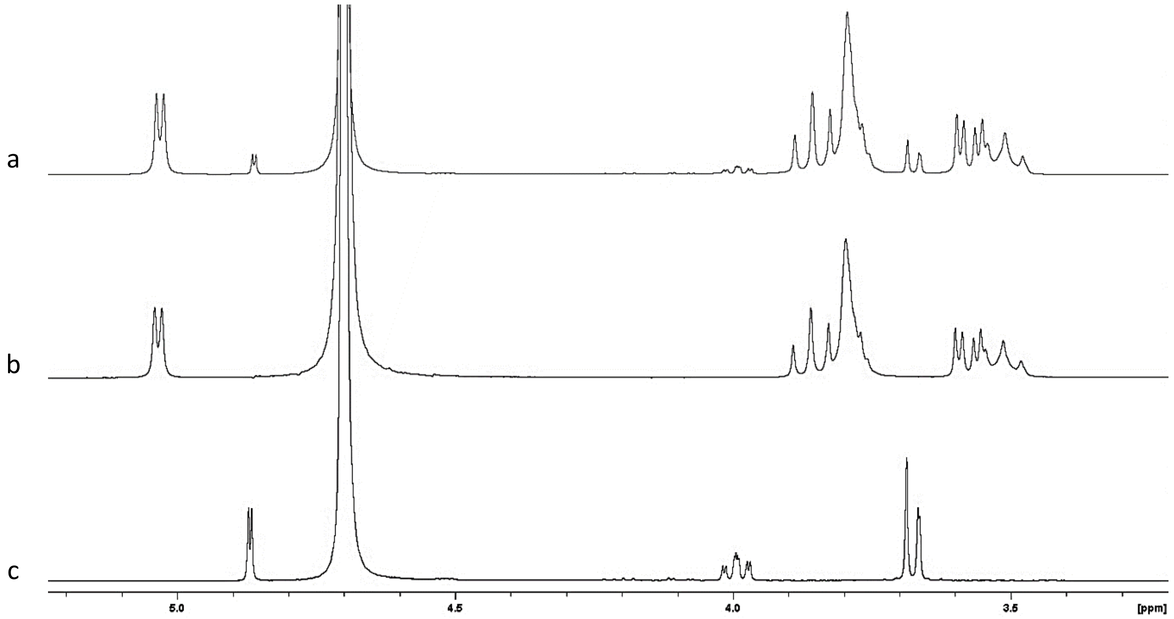

Figure S5 ^{1}H NMR spectra of (a) ascorbic acid, (b) γCD and (c) ascorbic acid/γCD inclusion complexes in D_2O.

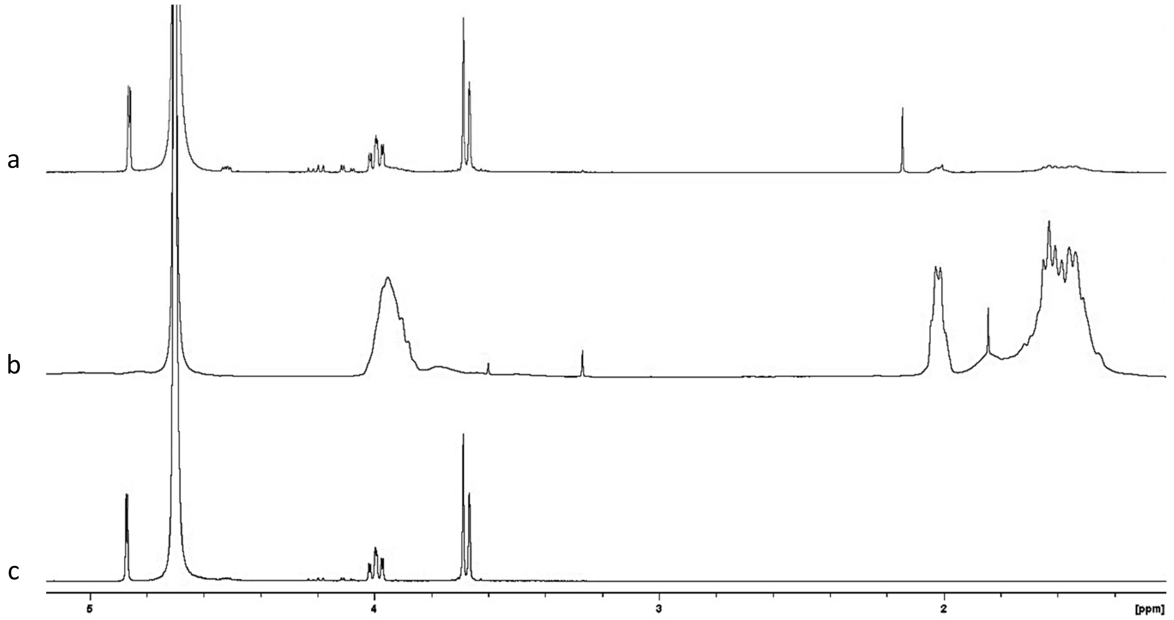

Figure S6 ^{1}H NMR spectra of (a) ascorbic acid, (b) PVA and (c) ascorbic acid/PVA inclusion complexes in D_2O.

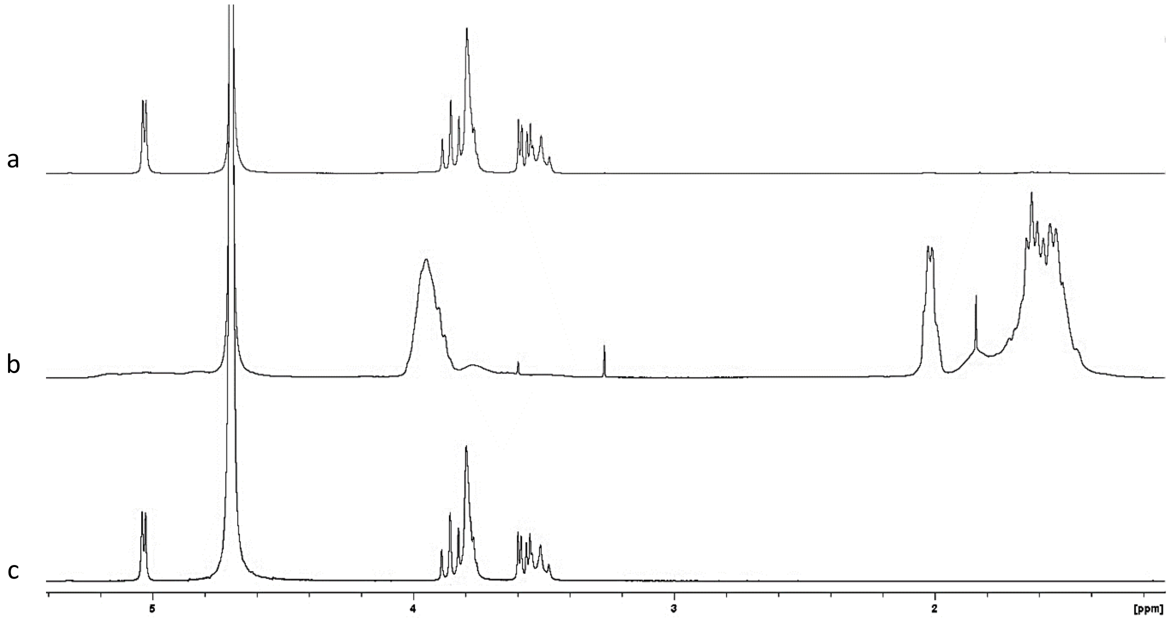

Figure S7 ^{1}H NMR spectra of (a) γCD, (b) PVA and (c) γCD/PVA inclusion complexes in D_2O.

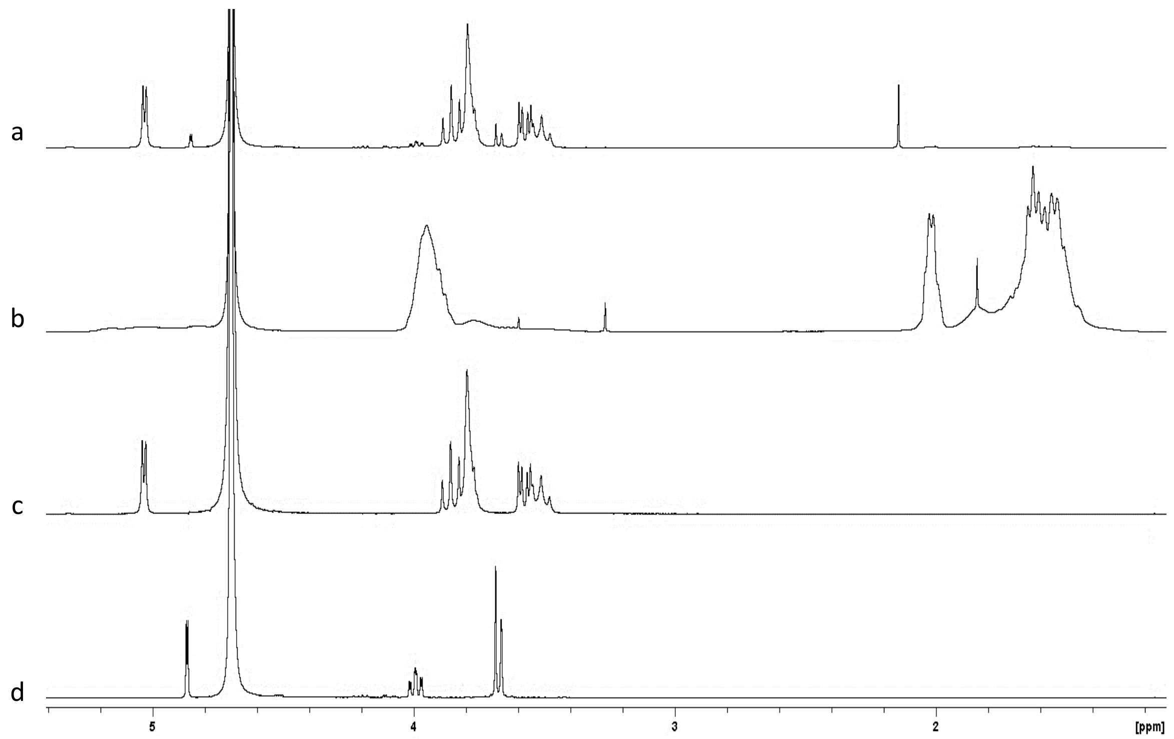

Figure S8 ^{1}H NMR spectra of (a) ascorbic acid, (b) γCD (c) PVA and (d) ascorbic acid/γCD/PVA complexes in D_2O.

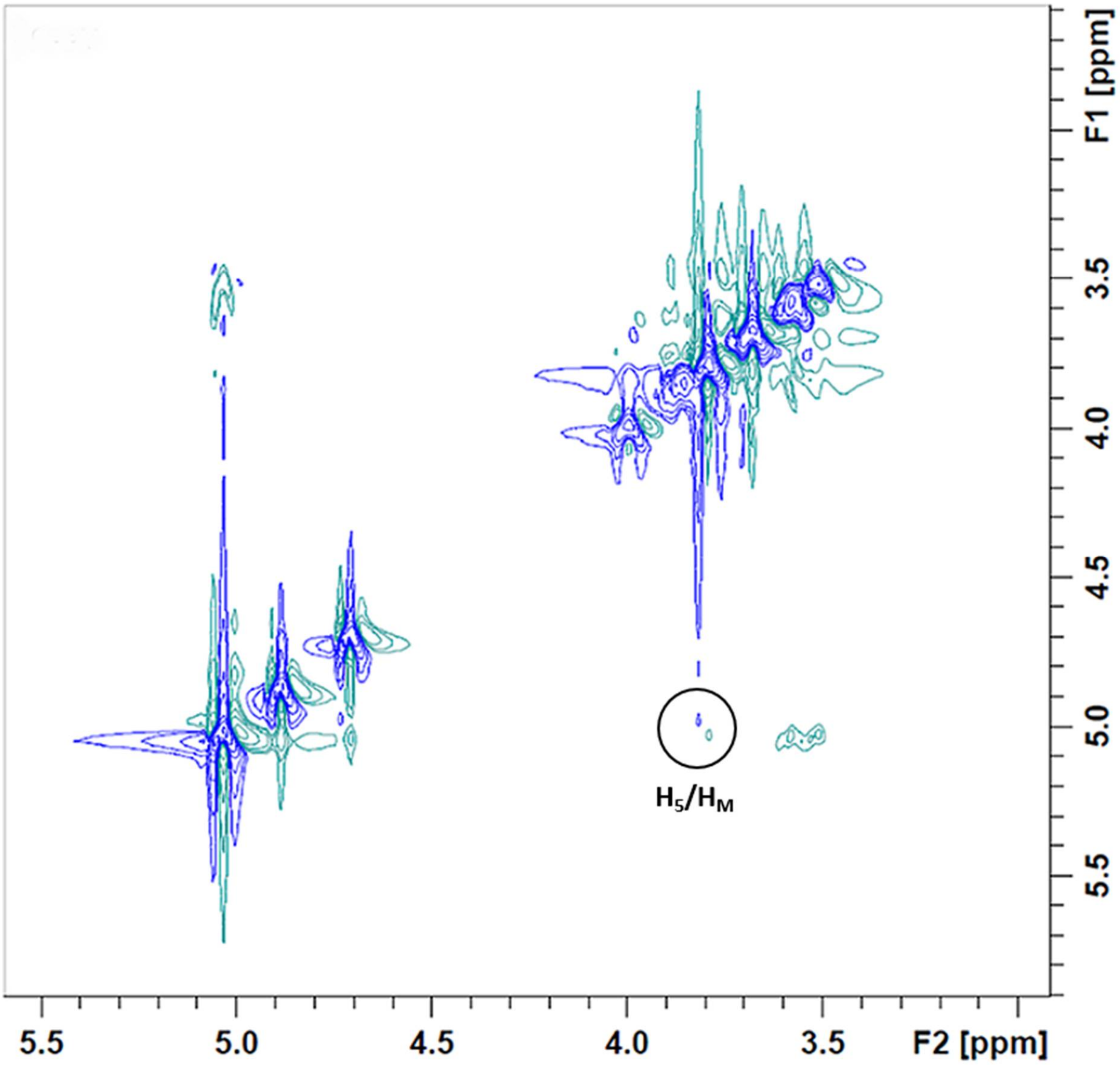

Figure S9 Rotating overhauser effect spectroscopy (ROESY~~ROSEY~~) pattern of ascorbic acid/γCD complexes.

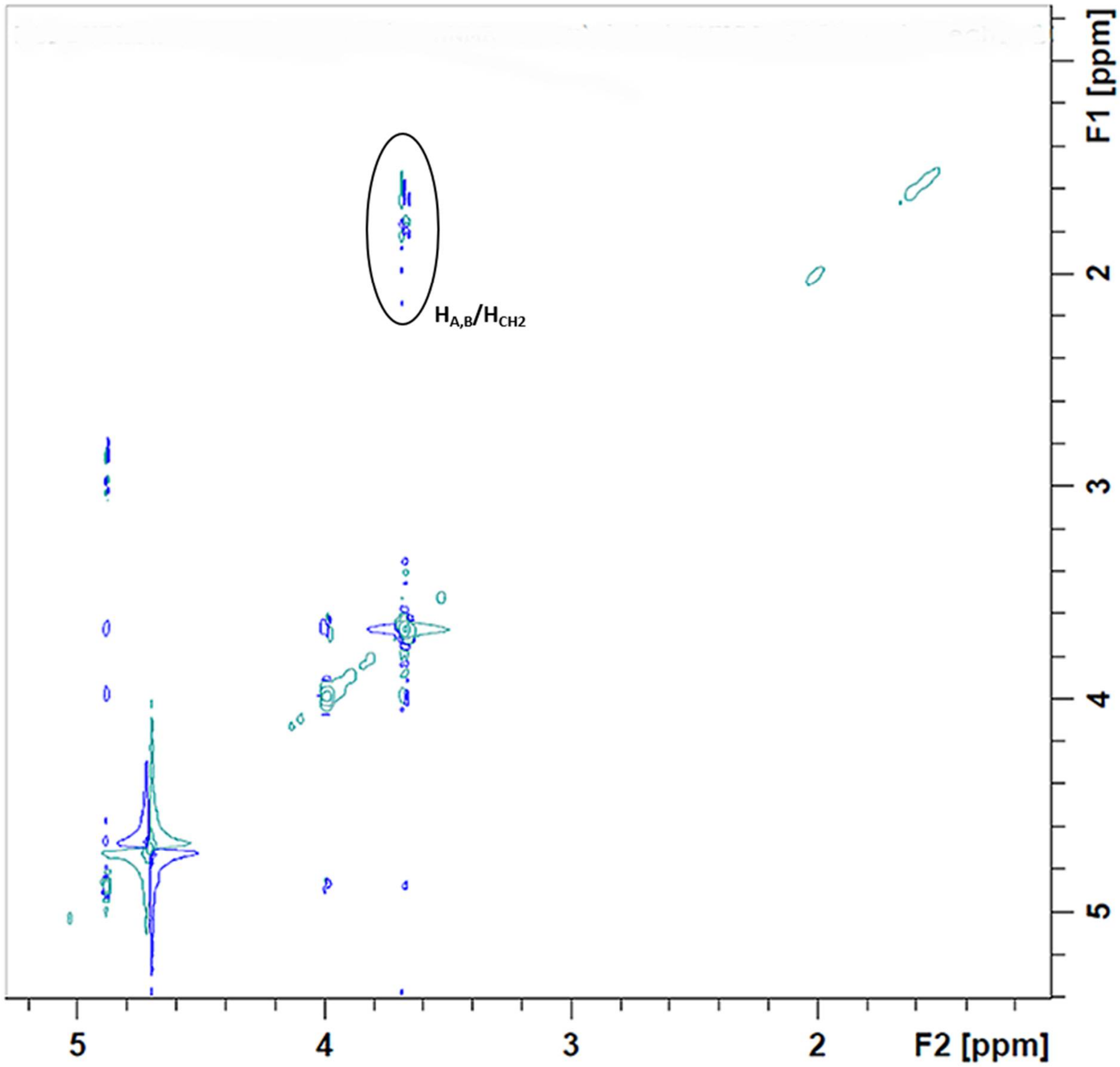

Figure S10 Rotating overhauser effect spectroscopy (~~ROSEY~~ROESY) pattern of ascorbic acid/PVA complexes.

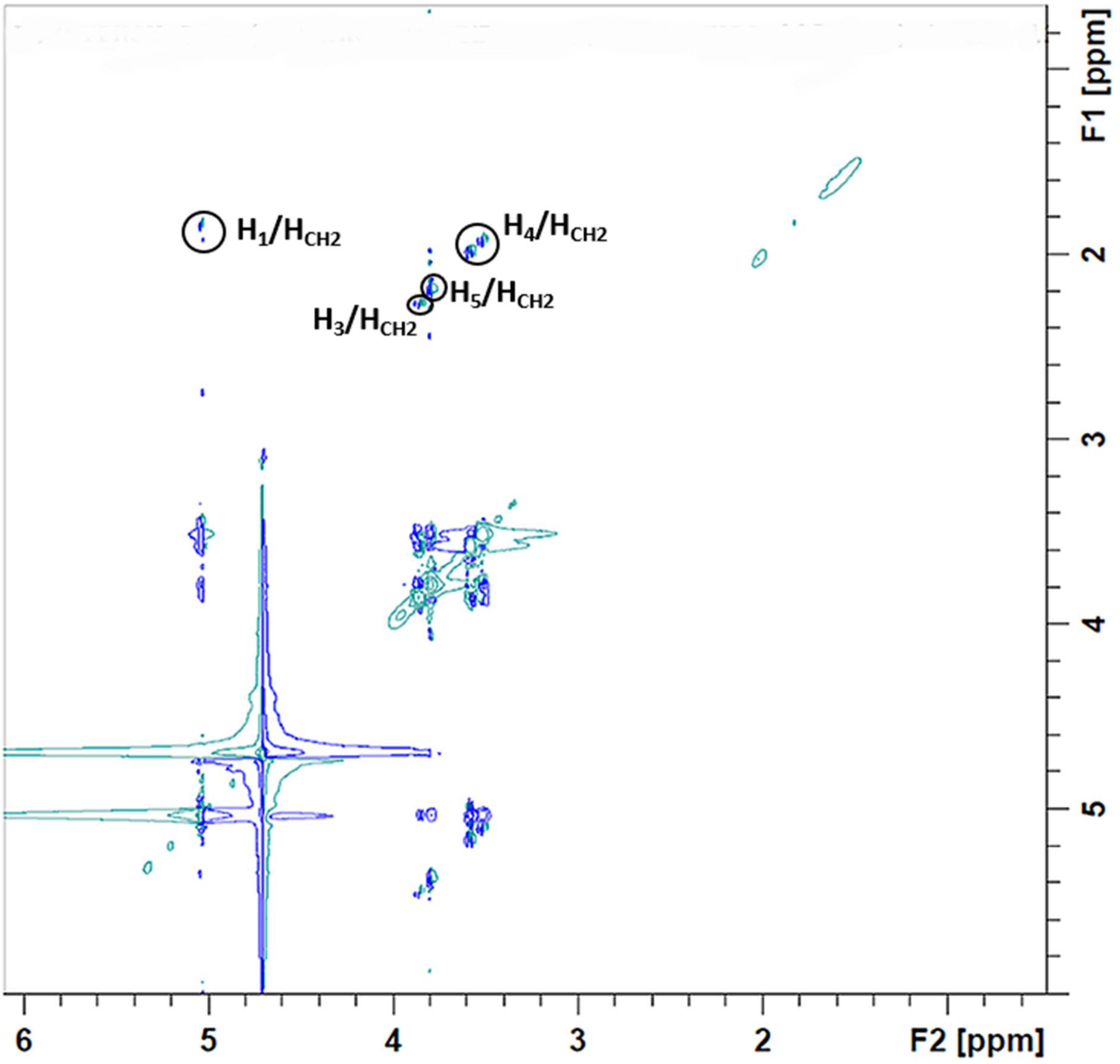

Figure S11 Rotating overhauser effect spectroscopy (~~ROSEY~~ROESY) pattern of γCD/PVA complexes.

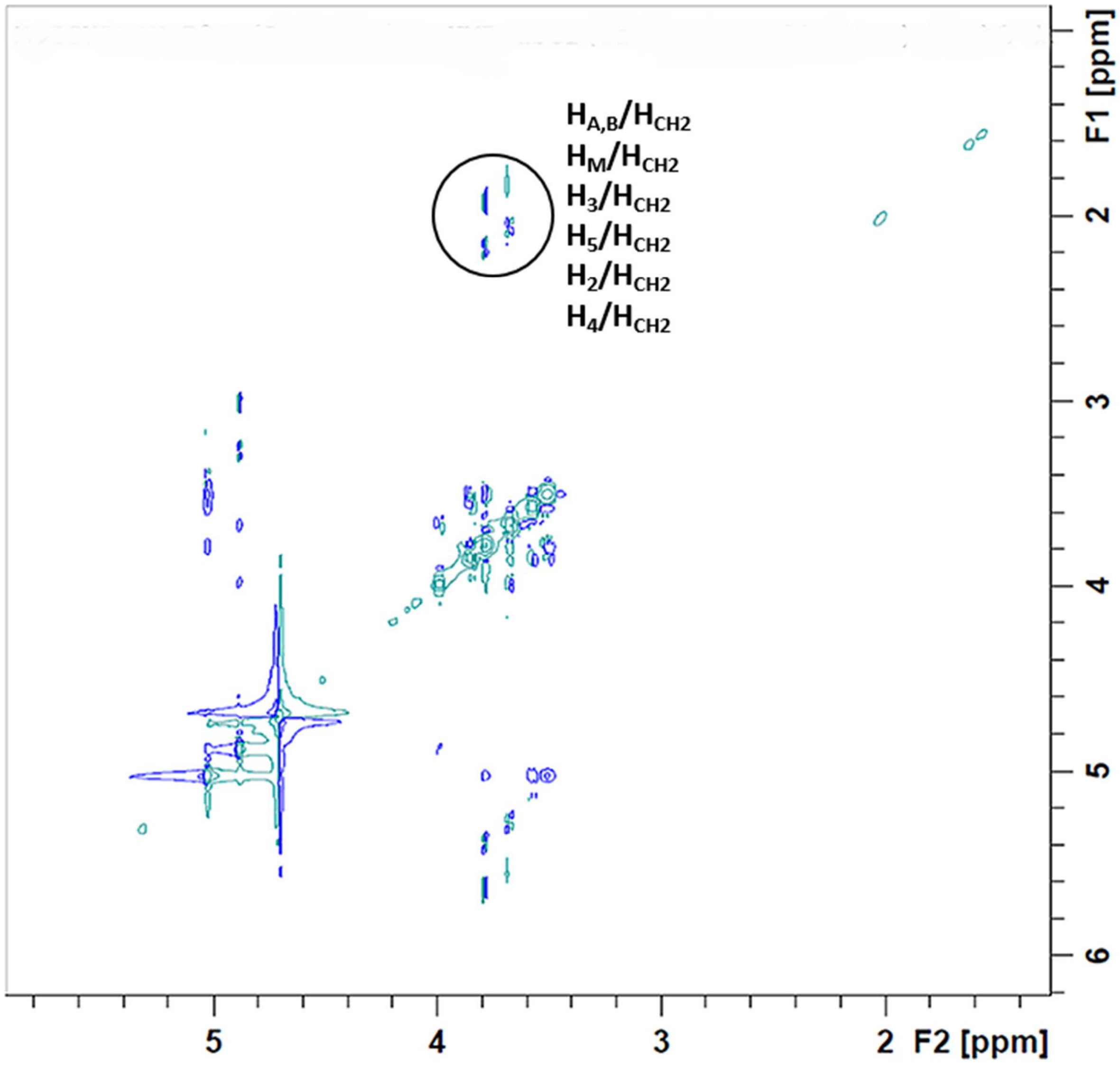

Figure S12 Rotating overhauser effect spectroscopy (ROESSEY) pattern of ascorbic acid/γCD/PVA ternary complexes.
